# Isolated congenital absence of a single pulmonary valve cusp

**DOI:** 10.1016/j.xjtc.2021.07.021

**Published:** 2021-08-04

**Authors:** Shinya Inoue, Atsuo Mori, Yasunori Iida, Hidetoshi Oka

**Affiliations:** a Department of Cardiovascular Surgery, Kawasaki Municipal Hospital, Kawasaki, Kanagawa, Japan; b Department of Cardiovascular Surgery, Saiseikai Yokohamashi Tobu Hospital, Yokohama, Kanagawa, Japan

**Figure:**
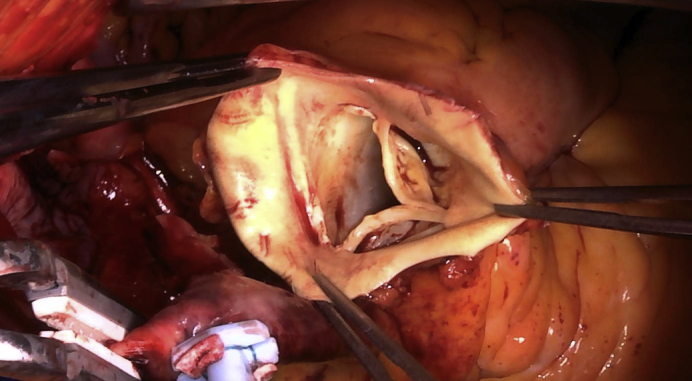
Examination of the pulmonary valve reveals absence of the posterior nonfacing leaflet.

**Central Message**

Congenital absence of 1 cusp of the pulmonary valve without any rudimentary tissue or nubbin of tissue at the annulus was associated with the other 2 cusps of normal size and morphology.

See Commentaries on pages 437 and 439.

Congenital absence of the pulmonary valve (PV) is a rare cardiac anomaly usually associated with ventricular septal defect and an infundibular or obstructive PV annulus (so-called tetralogy with absent PV). Almost all cases of congenital absence of the PV cusp are associated with other congenital heart defects. Occurrence as an isolated malformation, as in this case, appears extremely unusual. A literature review revealed 1 report of 2 normal pulmonary cusps and 1 absent cusp.^1^ Other reports have variously described a case of absent PV leaflet with another hypoplastic leaflet and a normal third leaflet,^2^ a case of complete absence of 1 leaflet with moderate hypoplasia of the other 2 leaflets,^3^ and a case of complete absence of 1 leaflet, with 1 normal cusp and 1 dysplastic cusp.^4^ This is therefore only the second report of congenital absence of a single cusp of the PV with normalcy of the other 2 cusps.

## Case Report

A 66-year-old Japanese woman was hospitalized due to heart failure. She presented with palpitations, shortness of breath, decreased urine output, leg edema, and orthopnea. She had been diagnosed with dilatation of the pulmonary artery (PA) during childhood, but the details were unknown. She had been hospitalized due to heart failure at age 50 years, but details of that hospitalization were unknown. Since then, she has been evaluated on an outpatient basis.

Blood pressure was 106/62 mm Hg and heart rate was 92 beats per minute. A grade 3/6, medium-pitch, systolic murmur at 3 left sternal border and grade 3/6, medium-pitch, diastolic murmur at 2 left sternal border were heard on chest auscultation. Peripheral oxygen saturation using pulse oximetry in room air was 89%.

**Figure 1:**
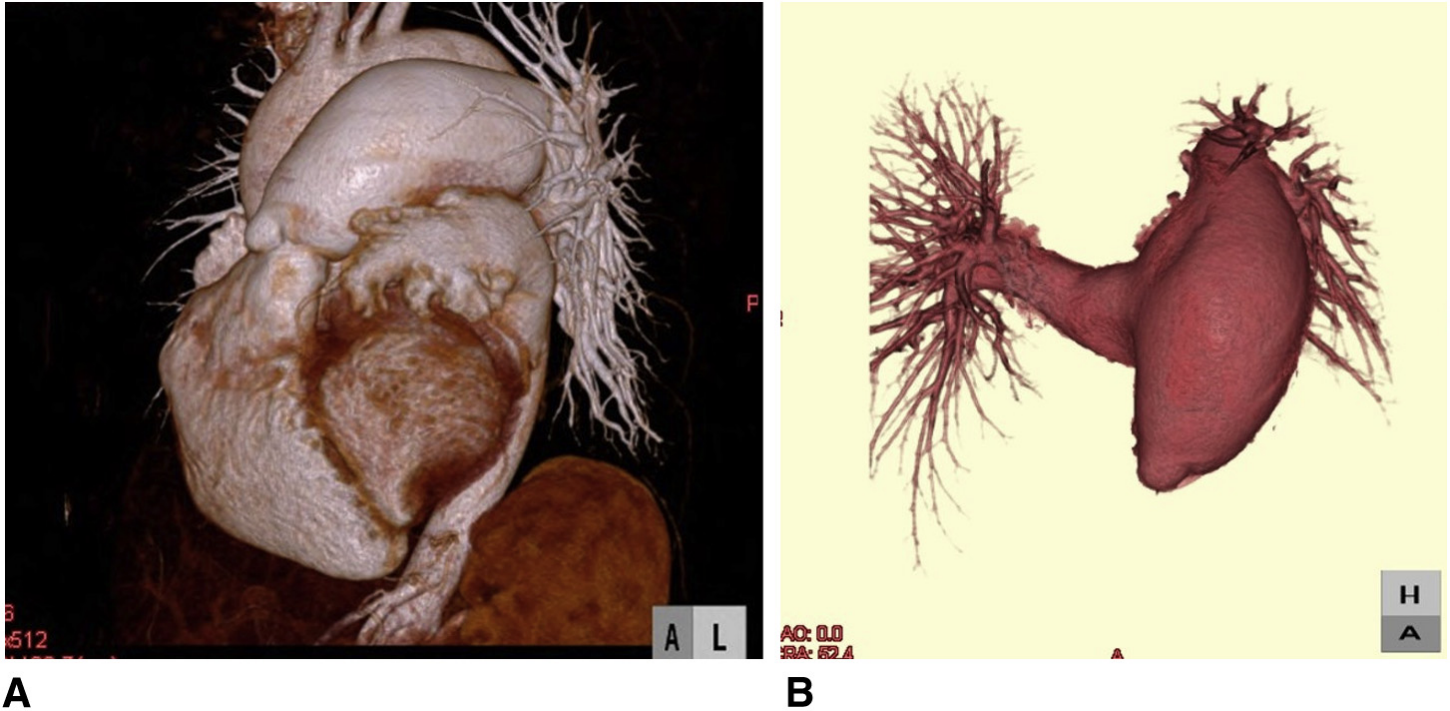
A and B, Preoperative computed tomography. The main pulmonary artery, right pulmonary artery, and left pulmonary artery are dilated.

Electrocardiography showed atrial fibrillation and complete right bundle branch block. Echocardiography showed an enlarged right ventricle, marked dilatation of the PA, moderate pulmonary regurgitation, and severe mitral regurgitation (deterioration from mild regurgitation). Left ventricular function was mildly depressed. In addition, the patient was diagnosed with hyperthyroidism. Computed tomography showed dilatation of the main PA, right PA, and left PA (Figure 1, *A* and *B*).

Cardiac catheterization showed normal PA pressure and absence of intracardiac shunting. PA systolic pressure was 22 mm Hg, PA diastolic pressure was 3 mm Hg, and mean PA pressure was 14 mm Hg. Angiocardiography confirmed the echocardiographic findings of PA dilatation, pulmonary regurgitation, and severe mitral regurgitation. The patient was therefore scheduled to undergo mitral valve replacement and reduction of the diameter of the PA.

**Figure 2:**
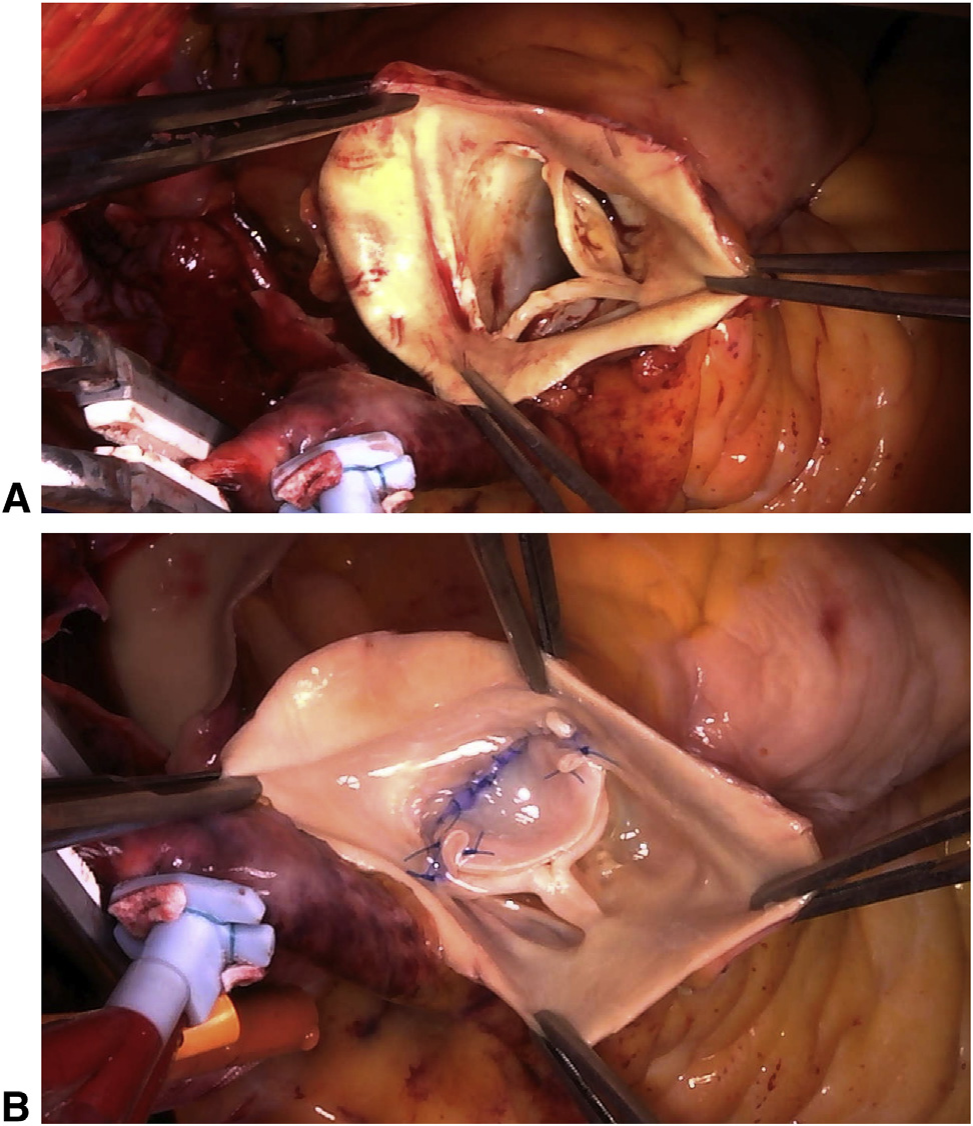
Intraoperative findings. A, Examination of the pulmonary valve reveals a normal right-facing leaflet, normal left lateral-facing leaflet, and absence of the posterior nonfacing leaflet without any rudimentary tissue or nubbin of tissue at the annulus. B, A trapezoid-shaped cut bovine pericardium graft is sutured to the annulus of the posterior nonfacing cusp, creating a new valve leaflet with adequate redundancy and reservoir during diastole.

Surgery was performed when she was aged 66 years. Intraoperatively, dilatations of the right ventricle and pulmonary trunk were noted. First, mitral valve replacement was performed using a mechanical 25-mm valve (Carbomedics, Inc, Austin, Tex). Evaluation of the mitral valve revealed no chordal rupture or elongation, and thus no prolapse. The mitral valve leaflets were degenerated and the left ventricle was dilated. Tethering was believed to be the cause of mitral regurgitation. Next, the main PA was transected along its entire circumference. Examination of the PV revealed a normal right-facing leaflet, normal left lateral-facing leaflet, and absence of the posterior nonfacing leaflet (Figure 2, *A*, and Video 1). No endocarditis was evident. The PV annulus measured approximately 21 mm in diameter. Confirming isolated absence of the posterior leaflet, a trapezoid-shaped cut bovine pericardial graft was sutured to the annulus of the posterior nonfacing cusp, to create a new valve leaflet, with adequate redundancy and reservoir during diastole (Figure 2, *B*, and Video 1). Trapezoid-shaped cuts were made on the anterior walls of the right, left, and main PAs, then sutured to reduce the diameter of the PA. Intraoperative transesophageal echocardiography performed after the repair revealed good coaptation of the PV leaflets and trivial regurgitation.

**Video 1:**
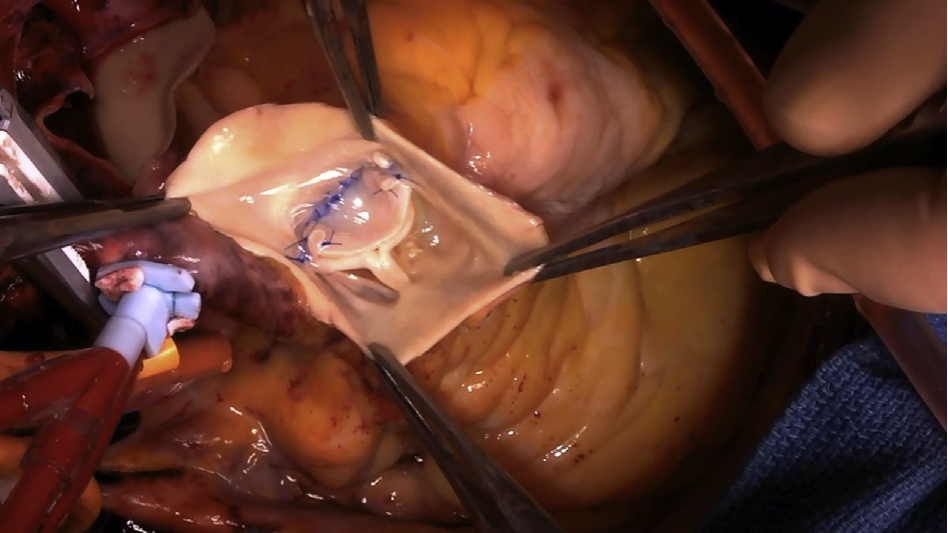
Inspection of the pulmonary valve reveals a normal right-facing leaflet, normal left lateral-facing leaflet, and absence of the posterior nonfacing leaflet without any rudimentary tissue or nubbin of tissue at the annulus. Semicircular-shaped cut bovine pericardium graft is sutured to the annulus of the posterior nonfacing cusp, creating a new valve leaflet with adequate redundancy and reservoir during diastole. Video available at: https://www.jtcvs.org/article/S2666-2507(21)00501-0/fulltext.

**Figure 3:**
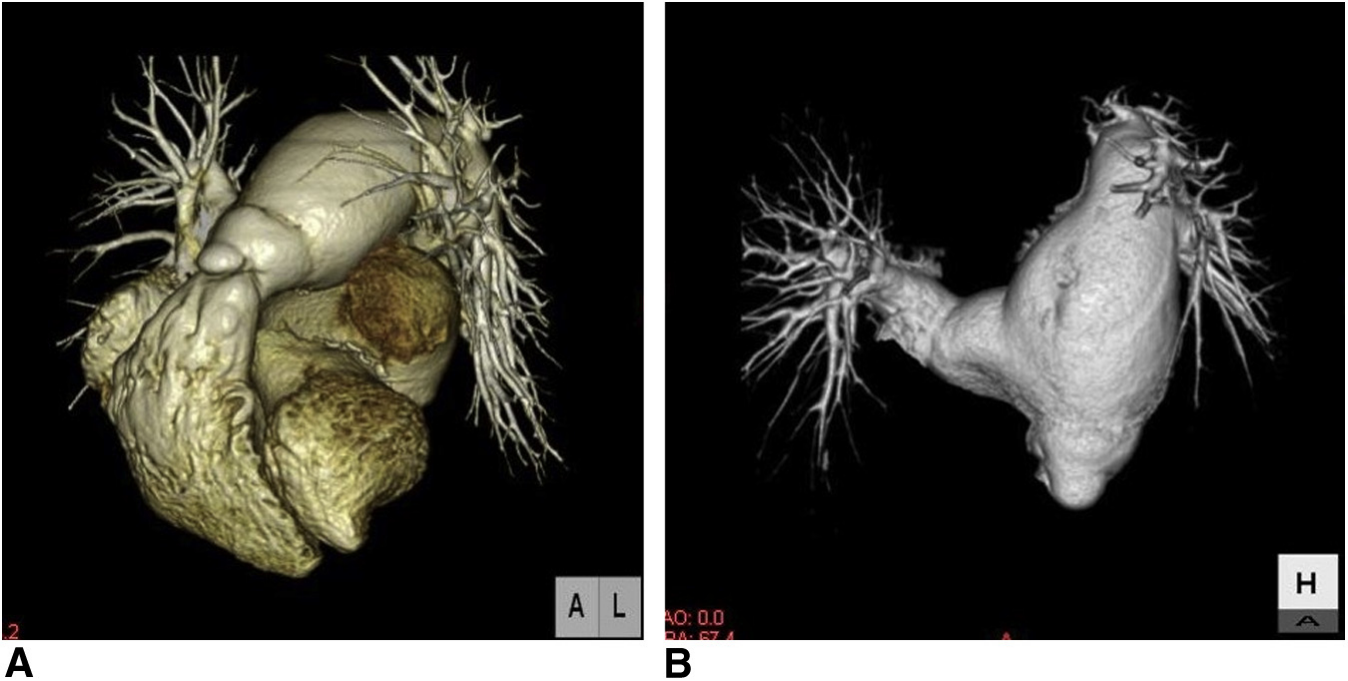
A and B, Postoperative computed tomography. A reduction is indicated in diameter of the main pulmonary artery to 52 mm, from the preoperative value of 66 mm.

Echocardiographic evaluation 1 week postoperatively indicated no pericardial effusion, normal functioning of the mechanical mitral valve, no pulmonary stenosis, and mild pulmonary regurgitation. The diameter of the main PA was 35 mm. Contrast-enhanced computed tomography performed 1 week postoperatively indicated a reduction in diameter of the main PA to 52 mm, from the preoperative value of 66 mm (Figure 3, *A* and *B*). Reduction of left atrial volume was also noted. Repeat echocardiography 1 year later showed a dilated right ventricle with slightly depressed systolic function and dilatation of the pulmonary trunk. The PV leaflets showed good mobility. No pulmonary stenosis was evident and only mild pulmonary insufficiency was present. The mechanical mitral valve was functioning well and the patient remained clinically asymptomatic.

Our institution's institutional review board policy does not deem it necessary to receive approval for cases that describe 1 patient. Written informed consent was obtained for publication.

## Discussion

Congenital absence of the PV is a rare but well-documented entity that is often characterized by a narrowed pulmonic annulus with rudimentary cusps, PA dilatation, and a mal-aligned ventricular septal defect. This constellation of cardiac defects is often referred to as tetralogy of Fallot with absent PV. Less often, an absent PV has been reported with other cardiac defects, including atrial septal defect, patent ductus arteriosus, or both. In such cases, PV anatomy usually consists of rudimentary remnants of all 3 leaflets. In our case, she showed no other congenital heart malformations, making this only the second report of congenital absence of a single cusp of the PV associated with normal structure of the remaining 2 cusps.

In 1997, Westaby and Katsumata^1^ documented a case of congenital absence of a PV leaflet with atrial septal defect. That was the only report before our own in which 2 pulmonary cusps were of normal size and morphology, whereas the third cusp was absent, without any rudimentary tissue or nubbin of tissue at the annulus. Westaby and Katsumata^1^ used a monocusp aortic homograft to repair the valve. A literature review by Sayger and colleagues^2^ revealed 1 report describing absence of a single PV leaflet with 1 hypoplastic leaflet and 1 normal leaflet. Cardiac anatomy and function were otherwise normal. They created a valve cusp from the posterior wall of the PA at the base of the pulmonary trunk. In 2015, Yamazaki and colleagues^3^ reported a case of complete absence of 1 leaflet with moderate hypoplasia of the other 2, together with dextrocardia. The patient had a patent foramen ovale and patent ductus arteriosus, although these closed spontaneously before age 7 years. PV replacement using a composite biologic valved conduit was performed. In 2015, Nassar and Anderson^4^ reported a case of complete absence of 1 leaflet, together with 1 normal cusp and 1 dysplastic cusp. Valve cusp reconstruction was performed using autologous tissue from the PA wall. Repair procedures thus varied among previous cases.

Ours was a rare case in that the patient had no other congenital cardiac malformations, and had thus remained asymptomatic until middle age, and also for the fact that congenital absence of 1 cusp of the PV was associated with normalcy of the other 2 cusps.

Our patient underwent PV plasty using a bovine pericardial patch. Follow-up revealed good mobility of the PV leaflets, no pulmonary stenosis, and only mild pulmonary insufficiency. She remains asymptomatic.

## Supplementary Data

Video 1 Inspection of the pulmonary valve reveals a normal right-facing leaflet, normal left lateral-facing leaflet, and absence of the posterior nonfacing leaflet without any rudimentary tissue or nubbin of tissue at the annulus. Semicircular-shaped cut bovine pericardium graft is sutured to the annulus of the posterior nonfacing cusp, creating a new valve leaflet with adequate redundancy and reservoir during diastole. Video available at: https://www.jtcvs.org/article/S2666-2507(21)00501-0/fulltext.
